# Editorial: One Health surveillance in practice: experiences of integration among human health, animal health, environmental health, and food safety sectors

**DOI:** 10.3389/fpubh.2024.1384988

**Published:** 2024-03-01

**Authors:** Guido Benedetti, Nadia Boisen, Joaquin M. Prada

**Affiliations:** ^1^Department of Infectious Disease Epidemiology and Prevention, Statens Serum Institut, Copenhagen, Denmark; ^2^Department of Bacteria, Parasites and Fungi, Statens Serum Institut, Copenhagen, Denmark; ^3^School of Veterinary Medicine, Faculty of Health and Medical Sciences, University of Surrey, Guildford, United Kingdom

**Keywords:** One Health, surveillance, human health, animal health, environmental health, food safety

Recognizing that human and animal health are interconnected brings along the challenge of integrating their respective health systems, including routine disease surveillance, outbreak management, and emergency preparedness. However, approaches in these different sectors are still unaligned in many ways, including their respective agendas, both at country and supranational levels. Since the early 2000s, the World Health Organization (WHO), the Food and Agricultural Organization of the United Nations (FAO), and the World Organization of Animal Health (WOAH) paved the road of multi-sectorial One Health (OH) approaches and collaborations, leading to the publication of the Tripartite Zoonoses Guide. Recently, the “One Health European Joint Programme” fostered cooperation in OH practice within and across European countries (1). Furthermore, various scientific networks and consortia have been set up to bring together professionals and experiences from different sectors.

Integration is key to the OH agenda, and to the challenge of preparedness and response to endemic diseases and other emerging threats. This Research Topic gathered first-hand, successful, and inspirational experiences about the integration of approaches, procedures and methodologies for OH surveillance across the human health, animal health, environmental health, and food safety sectors, at the local, national, or supranational levels.

To integrate existing surveillance systems effectively, the OH-EpiCap tool plays an important role by offering a semi-quantitative evaluation of “One-Healthiness”. Tegegne et al. developed this tool to strengthen OH surveillance systems, focusing on assessing their organization, operations and outputs. The tool is applicable to any disease surveillance system of OH relevance. The OH-EpiCap tool necessitates stakeholders' recognition of the importance of assessing their systems. Further, Moura, Collineau et al. measured the perceptions of users of the OH-EpiCap tool when applied to various national antimicrobial resistance (AMR) surveillance systems. They described the OH-EpiCap functionality, emphasizing its user-friendly application, comprehensive coverage of previously overlooked elements (such as the impact of integrated surveillance), and its focus on the governance of OH surveillance. The application of the OH-EpiCap tool was further explored with the Danish Integrated Antimicrobial Resistance Monitoring and Research Program for AMR and antimicrobial use in animals and humans (Moura, Høg et al.).

Introducing the OH perspective to disease surveillance entails recognizing how changing existing systems might affect stakeholders. Hence, the importance of collaboration among the participating actors. This was the focus of a qualitative evaluation of collaborations among AMR surveillance programmes in France by Bourély et al.. The study found that collaborations were mainly created through good personal relations between individuals in different sectors/areas of the programmes, who worked together due to personal interest, rather than due to the structure of the system. On the other hand, the mapping of the stakeholders and processes to integrate OH surveillance is a valuable exercise. Through questionnaires, data mapping and case studies, Amato et al.  illustrated the feasibility of mapping OH-relevant foodborne pathogen surveillance across human health, animal health, and food safety sectors in European countries. This adaptable methodology facilitates integration and underscores the need to transcend silo thinking for OH success, stressing the need for broader collaboration. Avila et al. further showed the importance of a holistic approach to disease surveillance, in a multidisciplinary and inclusive OH perspective by evaluating the presence of *Toxocara* spp. in public squares and parks in San Juan province, Argentina. Identifying zoonotic parasites with infection potential for humans in urban areas underscores the necessity of integrating expertise among different sectors. Addressing public health threat at the human-veterinary interface through “collaboration, communication, and coordination”, for positive health outcome in both humans and animals is key. This study by Le Bouquin et al. presents a routine surveillance system that brings together the human and veterinary sectors for the emerging zoonoses botulism in France, expanding the focus from farmed animals, which are usually under the OH spotlight, to wild animals and the entire ecosystem.

To design, implement, and evaluate integrated surveillance systems, Rivers et al. developed a framework focusing on output-based standards, with an emphasis on zoonotic threats, following the case of *Echinococcus multilocularis* in Great Britain. Defining objectives for such a system is important, and depends on the hazard—whether it is an endemic disease or a potential new introduction. Additionally, quantifying and communicating uncertainty, especially to non-technical audiences, can be challenging.

An understanding of laboratory methods is important to successful integration of surveillance systems. However, existing schemes often target single sectors, while cross-sectoral panels are key to OH. Tast Lahti et al. evaluated European laboratories' cross-sectoral proficiency for foodborne pathogens *Campylobacter* spp., *Salmonella* spp. and *Yersinia enterocolitica*, informing future proficiency tests and external quality assessments in OH. Takeaways emphasized the critical importance of well-defined targets and robust characterization methods for effective pathogen detection. These schemes foster international collaborations, which are pivotal to outbreak investigations and standardization. Future assessments should integrate genomic analysis to advance foodborne zoonosis methodologies.

Setting up an information system for genomic surveillance is challenging, as highlighted by Knijn et al. in their development and application of the IRIDA-ARIES infrastructure in Italy, but leads to better standardization processes of routine surveillance data. Imagining a OH-relevant surveillance system in Europe that is aligned with European agencies requirements is a step toward creating findable, accessible, interoperable, and reusable data (2). Indeed, the availability of diagnostics to identify hazards of interest is also important for any sustainable programme. In recent years, molecular techniques have been used more extensively to allow a better understanding of new, emerging threats. In this context, Cherchame et al. evaluated Salmonella enterica serovars and enhanced the open-access databases with 73 new genomes, including more reference genomes, which improves bioinformatics surveillance.

Finally, this Research Topic gathered the experiences from a multi-country OH foodborne outbreak simulation exercise as part of the One Health European Joint Programme (Alves et al.). The exercise put the functional response to a threat of OH relevance into practice across different sectors and across multiple countries in Europe. It focused on the countries' capacities and capabilities for outbreak preparedness, management and response and it remains a rich collection of pitfalls and opportunities that can serve as a benchmark for the participating countries and as an inspirational guide for others.

In the post-SARS-CoV-2 pandemic world, preparedness has become the new buzzword of public health practice and OH a never-failing “buzz-adjective”. Given its key role, it is high time for OH surveillance to become practice and routine.

## Author contributions

GB: Writing—original draft, Writing—review & editing. NB: Writing—original draft, Writing—review & editing. JP: Writing—original draft, Writing—review & editing.
